# Transcriptomic differences between male and female *Trachycarpus fortunei*

**DOI:** 10.1038/s41598-020-69107-7

**Published:** 2020-07-23

**Authors:** Xiao Feng, Zhao Yang, Wang Xiu-rong, Wang Ying

**Affiliations:** 1 College of Forestry, Guizhou University, Guiyang, 550025 Guizhou China; 2 Institute for Forest Resources & Environment of Guizhou, Guizhou University, Guiyang, 550025 Guizhou China; 3 Key Laboratory of Forest Cultivation in Plateau Mountain of Guizhou Province, Guizhou University, Guiyang, 550025 Guizhou China; 4 Key Laboratory of Plant Resource Conservation and Germplasm Innovation in Mountainous Region (Ministry of Education), Guizhou University, Guiyang, 550025 Guizhou China

**Keywords:** Plant breeding, Plant genetics

## Abstract

*Trachycarpus fortunei* (Hook.) is a typical dioecious plant, which has important economic value. There is currently no sex identification method for the early stages of *T. fortunei* growth. The aim of this study was to obtain expression and site differences between male and female *T. fortunei* transcriptomes. Using the Illumina sequencing platform, the transcriptomes of *T. fortunei* male and female plants were sequenced. By analyzing transcriptomic differences, the chromosomal helical binding protein (*CHD1*), serine/threonine protein kinase (*STPK*), cytochrome P450 716B1, and UPF0136 were found to be specifically expressed in *T. fortunei* males. After single nucleotide polymorphism (SNP) detection, a total of 12 male specific sites were found and the THUMP domain protein homologs were found to be male-biased expressed. Cytokinin dehydrogenase 6 (*CKX6*) was upregulated in male flowers and the lower concentrations of cytokinin (CTK) may be more conducive to male flower development. During new leaf growth, flavonoid and flavonol biosynthesis were initiated. Additionally, the flavonoids, 3′,5′-hydroxylase (*F3′5′H*), flavonoids 3′-hydroxylase, were upregulated, which may cause the pale yellow phenotype. Based on these data, it can be concluded that inter-sex differentially expressed genes (DEGs) and specific SNP loci may be associated with sex determination in *T. fortunei*.

## Introduction

*Trachycarpus fortunei* (Hook.) H. Wendl. (Fam.: *Trachycarpus*) are commonly known as "mountain palm" or "windmill palm" evergreen trees. Its flowers are unisexual and dioecious. *T. fortunei* is widely planted throughout China, where its leaf sheath fiber is often used as a rope and its unopened flower buds, also known as "brown fish," are edible and consumed^1^. *T. fortunei* is an important economic and landscaping plant. Plants of different genders often have different economic values; if seeds and fruits are used as harvesting objects, a large number of female plants are needed, while greening forests dominated by vegetative organs require male plants due to their higher economic value^2^. The sex of mature *T. fortunei* plants is generally identified by the inflorescence phenotype. The male upper inflorescence has 2–3 branches and the lower part is branched, while the female inflorescence has 4–5 conical branches and 6 staminodes, and often bears residual fruit in the inflorescences from the previous year. With the exception of different inflorescences and floral organs, other morphological signs of male and female *T. fortunei* do not exhibit obvious sexual dimorphism. *T. fortunei* also has adult non-inflorescence strains. The lack of inflorescence at the seedling stage leads to the lack of markers for the identification of male and female plants in the early stage of *T. fortunei*. Studying *T. fortunei* flower inflorescence and flower body development is a key factor for understanding the evolutionary relationship between the palm family and other angiosperm families^3^.

Current theories on plant sex determination concentrate on dioecious species^4^. The emergence of high-throughput sequencing technology and generation of large-scale data from non-model species has been a turning point in uncovering potential parthenogenetic development genes^5^. Transcriptomes are investigated by deep sequencing technology (i.e., RNA-Seq), which is essential for interpreting the functional elements of the genome, revealing the molecular components of cells and tissues, and understanding development and disease^6^. Analyzing transcriptomic differences in dioecious flower buds contributes to the screening of gender-related differentially expressed genes (DEGs), such as in studies on shrub willow^7^, *Quercus suber*^8^, Diospyros lotus^9^, and *Ginkgo biloba*^10^. Currently, no studies have investigated the relevant mechanisms of *T. fortunei* sex determination and only the screening of improved varieties during the introduction process has been conducted. In this study, RNA-Seq was used to provide a molecular basis for revealing differences in the transcriptional levels between *T. fortunei* male and female flowers and leaves. The findings of this study will enhance our understanding of the differences between male and female *T. fortunei*, facilitate the development of gender markers, and lay a foundation for furthering our understanding of *T. fortunei* sex determination and flower organ development.

## Results

### Raw data quality assessment and composition

In order to improve the quality and integrity of the assembly, 12 cDNA libraries were combined for assembly. Through the quality control of raw reads (Table 1), the Q20 ratio of all samples was > 97.56%, Q30 was > 93.82%, GC content ranged from 47.1 to 48.55%, and the unknown N base ratio was < 0.01%. The GC content of each sample was horizontally distributed, indicating that the sequencing quality was generally good and the data was reliable. Through the Trinity read assembly, a total of 431,753 transcripts and 158,533 unigenes were obtained. The N50 of transcripts and unigenes were 2,639 and 1,867 bp, respectively, indicating that the assembly integrity was relatively high. Raw data were uploaded to the NCBI/SRA database (accession Nos. SRR10120876–SRR10120887).

**Table 1:** Sample sequencing output data quality assessment form.

Sample ID	Read sum	Base sum	Q20	Q30	GC (%)	N (%)
Pt-ff1	44,913,516	6,737,027,400	97.67	94.04	47.56	0
Pt-ff2	48,017,968	7,202,695,200	97.63	93.96	48.55	0
Pt-ff3	48,645,406	7,296,810,900	97.67	94.06	47.26	0
Pt-fl1	48,517,790	7,277,668,500	97.8	94.31	47.22	0
Pt-fl2	48,059,210	7,208,881,500	97.7	94.12	47.5	0
Pt-fl3	47,985,230	7,197,784,500	97.73	94.16	47.4	0
Pt-mf1	50,344,970	7,551,745,500	97.67	94.06	47.42	0
Pt-mf2	48,395,086	7,259,262,900	97.62	93.91	47.8	0
Pt-mf3	49,530,474	7,429,571,100	97.64	94.14	47.5	0.01
Pt-ml1	44,413,774	6,662,066,100	97.72	94.15	47.1	0
Pt-ml2	46,870,966	7,030,644,900	97.56	93.82	47.59	0
Pt-ml3	48,786,860	7,318,029,000	97.67	94.04	47.26	0

### Functional comment

Unigenes were compared with the major databases (Table 2). A total of 69,074 (43.57%) unigenes were obtained from the aforementioned databases. The maximum number of annotated unigenes was obtained from the NCBI NR database (66,355 unigenes, 41.86%), and the minimum number was obtained from the GO database (13,511 unigenes, 8.52%).

**Table 2:** Unigene comment statistics.

Anno_Database	Annotated_Number	300 ≤ length < 1,000	Length ≥ 1,000
COG_Annotation	16,197	8,640	7,557
GO_Annotation	13,511	5,364	8,147
KEGG_Annotation	19,581	8,181	11,400
KOG_Annotation	35,348	18,853	16,495
Pfam_Annotation	24,446	12,406	12,040
Swissprot_Annotation	32,535	14,572	17,963
eggNOG_Annotation	52,992	27,298	25,694
nr_Annotation	66,355	37,197	29,158

### Gene expression analysis

**Figure 1:**
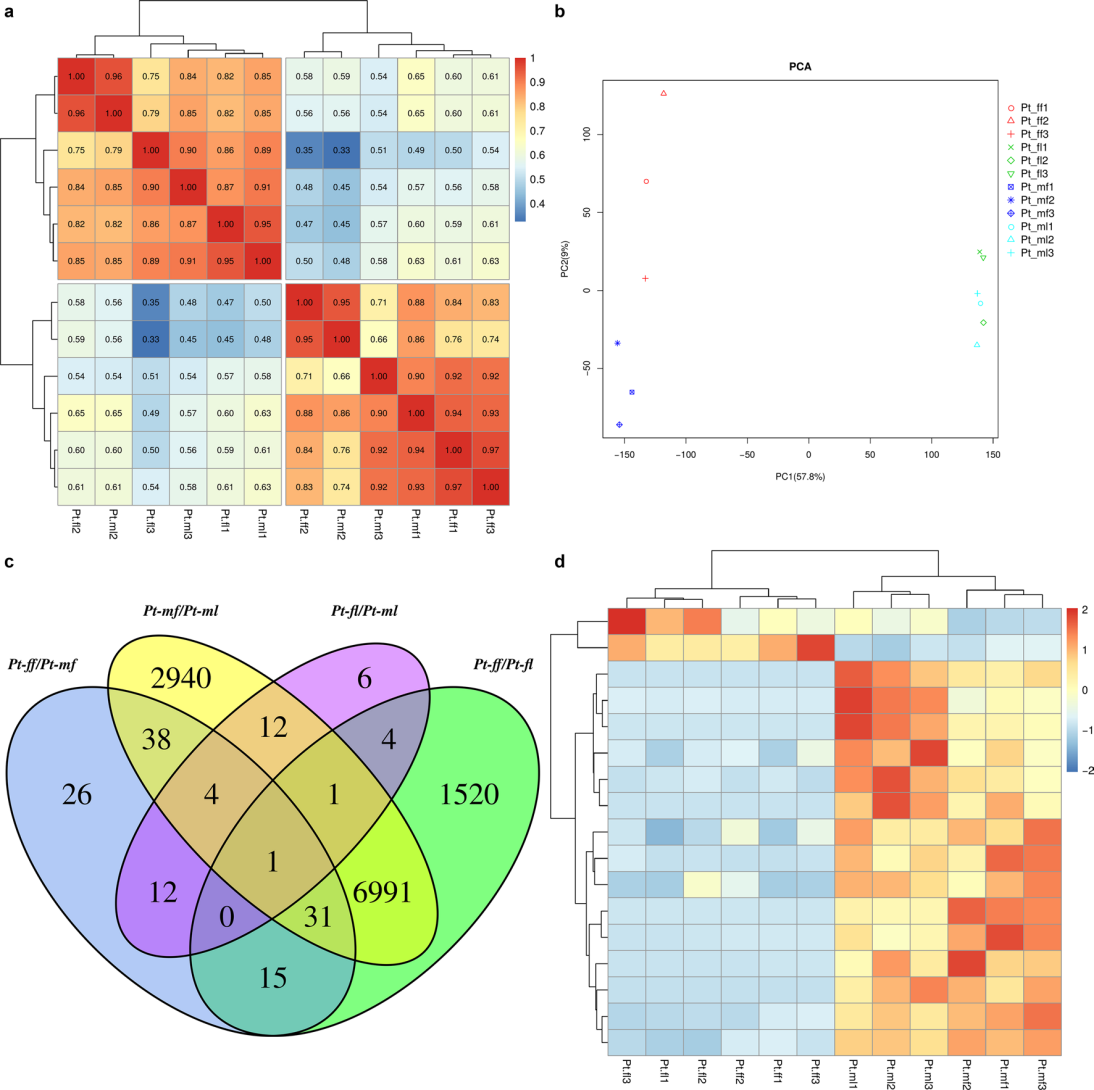
(**a**) Heatmaps of the expression levels between two samples; (**b**) PCA; (**c**) Comparison of Venn diagrams; (**d**) Heatmaps of DEGs in Pt-ff/Pt-mf and Pt-fl/Pt-ml.

Bowtie compared the sequenced reads with the unigene library by using the expected number of Fragments Per Kilobase of transcript sequence per Millions (FPKM) base pairs sequenced as the expression abundance of the corresponding unigenes. Pt-ff and Pt-mf were grouped together, Pt-ml and Pt-fl were grouped together, and flowers and leaves were distinguishable (Fig. 1a), wherein the value of PCA1 was 57.8% and the value of PCA2 was 9% (Fig. 1b).

### Functional enrichment analysis of DEGs

There were 127 DEGs between Pt-ff and Pt-mf (Fig. 1c), which consisted of 76 upregulated and 51 downregulated genes. Between Pt-fl and Pt-ml, 32 genes were upregulated and eight were downregulated. There were 17 DEGs in Pt-ff/Pt-mf and Pt-fl/Pt-ml (Fig. 1d). In Pt-ff/Pt-mf and Pt-fl/Pt-ml, 5 DEGs (i.e., TRINITY_DN56254_c0_g1, TRINITY_DN82812_c2_g2, TRINITY_DN70437_c2_g4, TRINITY_DN61762_c0_g1, and TRINITY_DN81704_c4_g4) were expressed in male plants. Additionally, 1 DEG, β-carotene 3-hydroxylase 2 (TRINITY_DN74609_c4_g1), was expressed in four combinations.

The GO enrichment analysis revealed that the Pt-ff/Pt-mf DEGs grouped into 56 categories, including transcriptional regulation, DNA-templated (GO: 0006355), sequence-specific DNA binding (GO: 0043565), and nucleus (GO: 0005634). Pt-fl/Pt-ml DEGs grouped into 19 categories, including cell wall (GO: 0005618), cell wall modification (GO: 0045545), and pectinesterase activity (GO: 0030599). The Pt-ff/Pt-ml DEGs grouped into 637 categories, including protein phosphorylation (GO: 0006468), regulation of transcription, DNA-templated (GO: 0006355), microtubule-based movement (GO: 0007018) and so on. The Pt-mf/Pt-fl DEGs grouped into 670 categories, including regulation of transcription, DNA-templated (GO: 0006355), protein phosphorylation (GO: 0006468), microtubule-based movement (GO: 0007018) and so on.

**Figure 2:**
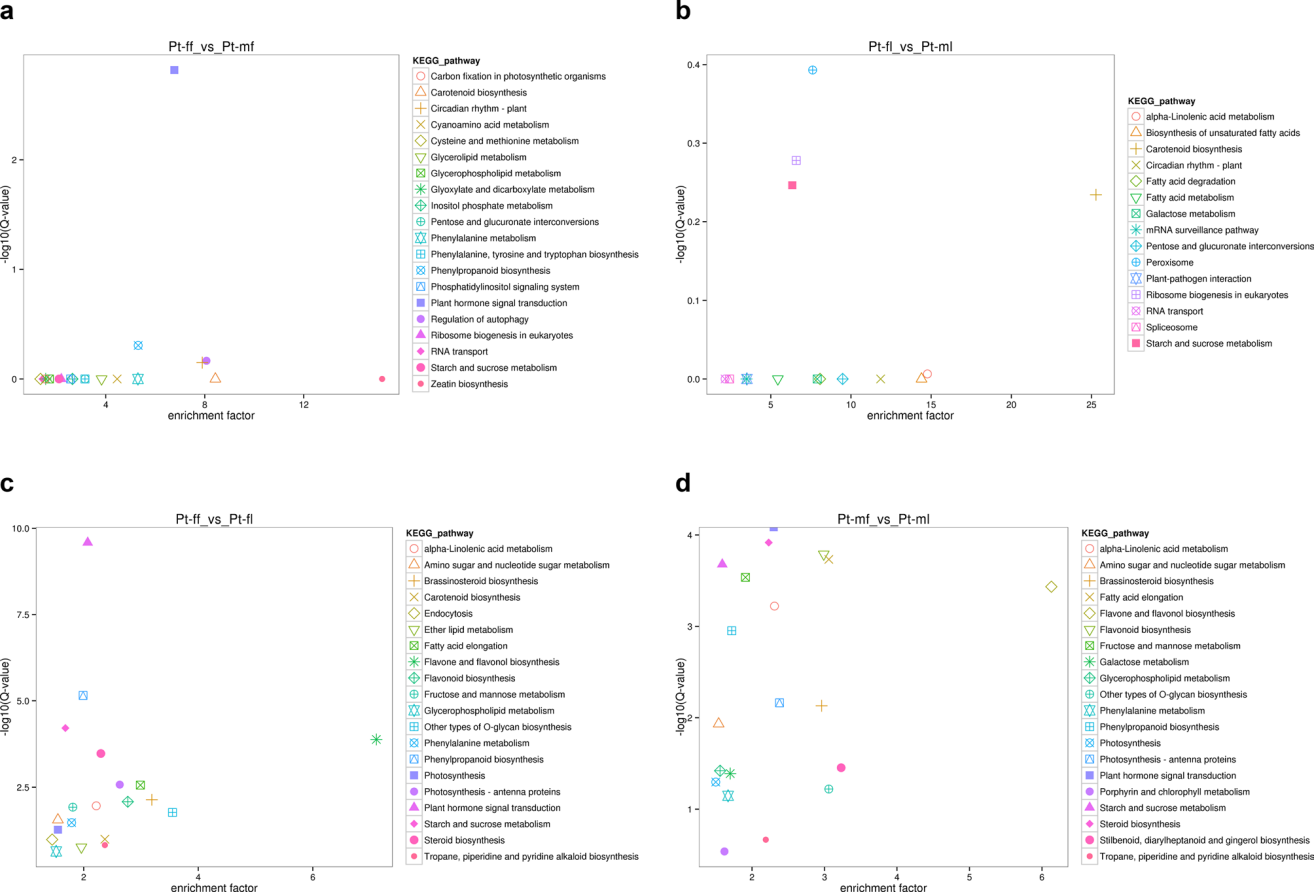
(**a**) Pt-ff/Pt-mf enrichment pathway factor map; (**b**) Pt-fl/Pt-ml enrichment pathway factor map; (**c**) Pt-ff/Pt-fl enrichment pathway factor map; (**d**) Pt-Mf/Pt-ml enrichment pathway factor map.

In Pt-ff/Pt-mf, the KEGG enrichment analysis and mapping of the DEGs (Fig. 2a) revealed that plant hormone signal transduction (ko04075) had the lowest q-value, indicating an obvious difference fold. Zeatin biosynthesis (ko00908) also had a large abscissa enrichment factor. In Pt-fl/Pt-ml (Fig. 2b), alpha-linolenic acid metabolism (ko00592), ribosome biogenesis in eukaryotes (ko03008), and starch and sucrose metabolism (ko00500), among other terms, had q-values that sequentially decreased. In Pt-ff/Pt-fl and Pt-mf/Pt-ml (Fig. 2c,d), the enrichment factors of flavonoid and flavonol biosynthesis (ko00944) were > 1.

### RT-qPCR verification

**Figure 3:**
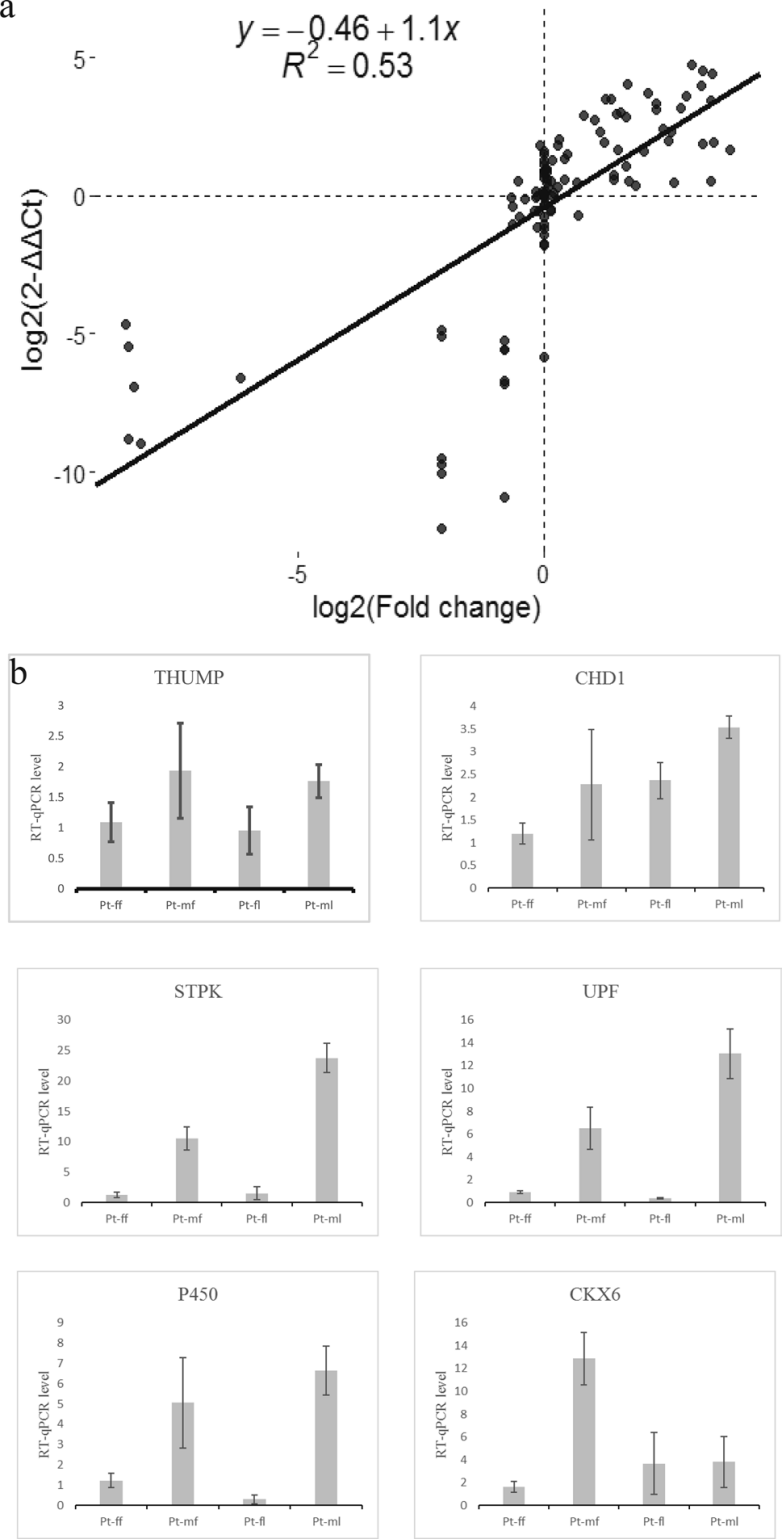
(**a**) The relative quantification of fluorescence (log_2_(2^−ΔΔCt^)) correlated with log_2_(fold change) linear regression. (**b**) Fluorescence relative quantification results (2^−ΔΔCt^) were calculated from the mean Pt-ff control (Ct).

Quantitative fluorescence analysis and RNA expression quantification were followed by linear regression analysis (Fig. 3a). Results revealed that the expression levels of STPK, UPF, and P450, which are specifically expressed in male plants, were significantly different compared with female plants; genes that were specifically expressed are presented (Fig. 3b). For example, the expression level of STPK in Pt-ml and Pt-mf was 23.76, which was 10.52 times that of Pt-ff.

### SNP full transcriptome detection

By searching transcriptomic single nucleotide polymorphisms (SNPs), 12 SNP loci in the male and female strains were identified with base substitutions and homozygous homology from the same sample (Table 3); these sites may be gender markers that distinguish the male sex in *T. fortunei*. It is worth noting that TRINITY_DN73064_c0_g1 is 1 DEG in Pt-ff/Pt-mf and Pt-fl/Pt-ml that was upregulated in male plants at the 1,066 site (5′–3′ locus). There is a base-switching event where the G nucleotide is fixed in *T. fortunei* females and A in *T. fortunei* males. SSR detection found that there was a double-base repeat event inside the TRINITY_DN73064_c0_g1 gene and the repeating unit was 34 times. Male and female plants have C ↔ A, G ↔ A, T ↔ C, and C ↔ T, among other base conversion events, at the same base sites of the remaining seven genes.

**Table 3:** Hypothetical sex-related genes in *T. fortunei*.

Gene ID	POS	Pt-ff1|Depth; Pt-ff2|Depth; Pt-ff3|Depth	Pt-fl1|Depth; Pt-fl2|Depth; Pt-fl3|Depth	Pt-mf1|Depth; Pt-mf2|Depth; Pt-mf3|Depth	Pt-ml1|Depth; Pt-ml2|Depth; Pt-ml3|Depth
TRINITY_DN69373_c0_g2	89	C|7; C|7; C|7	C|7; C|6; C|7	A|7; A|4; A|6	A|2; A|12; A|6
	143	G|20; G|7; G|20	G|7; G|7; G|10	A|9; A|5; A|7	A|3; A|11; A|7
TRINITY_DN70551_c5_g1	1762	T|24; T|22; T|23	T|26; T|47; T|20	C|76; C|45; C|69	C|69; C|70; C|69
TRINITY_DN73064_c0_g1	1,066	G|8; G|2; G|4	G|4; G|4; G|4	A|22; A|9; A|19	A|9; A|15; A|19
TRINITY_DN73101_c1_g3	728	C|28; C|15; C|24	C|5; C|3; C|5	T|24; T|27; T|6	T|4; T|2; T|6
	1,276	A|22; A|15; A|26	A|7; A|4; A|8	C|20; C|14; C|3	C|2; C|2; C|3
TRINITY_DN77827_c1_g1	1,395	C|23; C|11; C|12	C|9; C|19; C|7	T|28; T|17; T|4	T|3; T|6; T|4
TRINITY_DN82283_c0_g1	176	A|5; A|4; A|6	A|31; A|11; A|22	C|7; C|7; C|22	C|25; C|8; C|22
TRINITY_DN85795_c1_g1	1693	T|7; T|3; T|2	T|6; T|9; T|2	C|10; C3; C|23	C|20; C|2; C|23
	1805	A|7; A|3; A|2	A|10; A|10; A|6	G|11; G|5; G|23	G|20; G|7; G|23
	1901	A|5; A|3; A|4	A|6; A|4; A|5	C|11; C|3; C|11	C|9; C|2; C|11
TRINITY_DN87151_c2_g1	885	G|83; G|96; G|67	G|27; G|20; G|23	A|160; A|206; A|20	A|18; A|19; A|20

POS is the site of the unigene where the sequence-specific site was located (5′–3′).

## Discussion

There are 15,600 dioecious angiosperms in 987 genera and 175 families that account for 5–6% of the total species^11^. Early ascertaining the sex of seedlings can accelerate the artificial selection in breeding programs^12^. Sex determination is a major shift in the evolutionary history of angiosperms, as dioecious, sex-determining genes are usually located in the non-recombinant regions of sex chromosomes^13^. Next generation sequencing (NGS) technology is a widely used to study the promotion of sex determination in flowering plants. De novo RNA‐Seq transcriptome assembly and expression analysis can inform the investigation of gender determination in dioecious specie, exploring genome-wide sex-biased expression patterns in different species revealed a broad variation in the percentage of sex-biased genes (ranging from 2% of transcripts in *Littorina saxatilis* to 90% in *Drosophila melanogaster*), a study show that significantly more genes exhibited male-biased than female-biased expression in *Asparagus officinalis*^14^. Sex-biased expression was pervasive in floral tissue in *Populus balsamifera*, but nearly absent in leaf tissue^15^. Sex-specifically expressed genes may be derived from silencing the inhibition of related genes or the deletion of homologous genes in corresponding sex tissues. There are several scenarios for the origin of sex-biased genes, including single-locus antagonism, sexual antagonism plus gene duplication and duplication of sex-biased genes^16^. In this study, transcripts were constructed and assembled from *T. fortunei* female and male flowers and leaves to identify genes with sex-biased expression. Our study showed that more genes exhibited male-biased than female-biased expression in *T. fortunei.* Genes with sex-biased expression, often contribute largely to the expression of sexually dimorphic traits, SNPs calling from segregated populations of dioecious plant can help to identify the sex-associated SNPs and corresponding loci, five DEGs were polymorphic with common SNPs in each sex type in *Eucommia ulmoides*^17^. By searching transcriptomic SNPs, 12 SNP loci in the male and female strains were identified with base substitutions and homozygous homology from the same sample. S^4^U (4-thiouridine), a modified nucleoside, is located in the receptor arm and D arm of eubacterial and archaeal tRNA. S^4^U8 is synthesized by 4-thiouridine synthase (Thil) to stabilize the folding of tRNA and acts as a sensitive trigger for UV irradiation response mechanisms^18,19^. TRINITY_DN73064_c0_g1, the THUMP domain-containing protein 1 homolog, is a homologue with a THUMP domain and is one of the Thil constituent domains; it has a double base (CT) 34-unit repeat and a male-specific site. It is differentially expressed in both *T. fortunei* male and female plants; such polymorphic biased genes may be linked together in the non-recombinant region of sex determination regions (SDR)^20^.

*CHD1* (TRINITY_DN61762_c0_g1) is highly expressed in *T. fortunei* male plants. In a previous study, rice CHD1 was highly expressed in plant leaves and the number of cells in the leaves and stems of CHD1 mutant plants decreased, the surface epidermis increased, and the chlorophyll a/b content of leaves decreased^21^. *CHD1* has little difference in terms of exon and intron changes, and variations are mainly concentrated in the introns. Interestingly, it has been used as a marker for early sex identification in birds and poultry^22–24^. Protein kinases are a class of enzymes that use ATP to phosphorylate other proteins and play important roles in controlling many aspects of cellular life, and are divided into three major subclasses: receptor tyrosine kinase (RTK), serine/su protein kinase (STPK), and histidine kinase^25^. TRINITY_DN81704_c4_g4 acts as a STPK that is synergistic with cyclins and serves as an important cellular regulatory factor. In *Arabidopsis*, the gene encoding the STPK, *OXI1*, is induced in response to extensive H_2_O_2_ stimulation and serves as an important part of the signal transduction pathway that links several oxidative signals with downstream responses^26^. *STPK* are highly expressed in *T. fortunei* males compared to female flowers and leaves, suggesting that there may be differences in the stress transmission of oxidative signals between male and female plants. UPF0136 (TRINITY_DN70437_c2_g4) and cytochrome P450 716B1 (cytochrome P450 716B1-like) (TRINITY_DN82812_c2_g2) were also highly expressed in male plants, however, their specific functions have yet to be explored.

KEGG enrichment of the DEGs in Pt-ff/Pt-mf revealed that zeatin biosynthesis (ko00908) was enriched and cytokinin dehydrogenase 6 (TRINITY_DN64789_c0_g1) was upregulated 6.98 times higher in male flowers. Cytokinin oxidase/dehydrogenase (CKXs) catalyze the irreversible degradation of cytokinins^27^. During the development of male and female *T. fortunei* flowers, the IAA, Abscisic acid (ABA) and zeatin riboside (ZR) contents of male flowers are lower than female flowers in the corresponding period^28^. Transgenic overexpression of 6 CKX members in *Arabidopsis* transgenic plants increased the breakdown of cytokinin with a content equivalent to 30–45% of wild-type CTK content; the existence of CTK is essential for survival and a lack of CTK leads to reduced plant apical meristem and leaf primordium activities^29^. Most *Brassica napus BnCKXs* are highly expressed in reproductive organs, such as buds, flowers, or siliques^30^. TRINITY_DN64789_c0_g1 was highly expressed in male flowers, indicating that CKXs were upregulated in male flowers and that lower CTK concentrations may be more favorable for male flower development. This spatial expression pattern may be related to the different CTK functions.

In Pt-ff/Pt-fl and Pt-mf/Pt-ml, flavonoid and flavonol biosynthesis (ko00944) were both > 1. Flavonoid 3′,5′-hydroxylase (*F3′5′H*) is a member of the cell pigment P450 family and is the key enzyme for the synthesis of 3′,5′-hydroxylhydrochemical pigmentation^31^. Flavonoid 3′-hydroxylase (*F3′H*) is involved in flavonoid biosynthesis. The *F3′H* gene is expressed in different tissues such as roots, stems, leaves, flowers, etc., and can change the color of plant flowers or seed coats^32^. Flavonoids, carotenoids, and beetroot are the main flower pigments^33^. In flavonoids, orange and charone are yellow pigments; thus, flavonoids and flavonols are colorless or very light yellow^34^. The upward expression of *F3′5′H* and *F3′H* in *T. fortunei* new leaf growth indicates that these components may cause the light yellow leaf color phenotype.

## Conclusion

By analyzing the transcriptomic differences between *T. fortunei* female and male flowers and leaves, *CHD1*, *STPK*, cytochrome P450 716B1, UPF0136 and THUMP domain-containing protein 1 homolog were found to be highly expressed in *T. fortunei* males. Through SNP site detection, a total of 12 male and female specific sites were found. CKX6 exhibited increased expression in male flowers. Lower CTK concentrations may be more conducive to the development of male flowers. In the early stages of leaf growth, flavonoid and flavonol biosynthesis were initiated and the up-regulated expression of *F3′5′H* and *F3′H* may cause the pale yellow phenotyper of the leaf.

## Materials and methods

### Materials

Materials were collected from an artificially planted *T. fortunei* forest located in Guiding County, Guizhou Province, China. The test site belongs to the mid-subtropical monsoon humid climate. The soil in the forest is yellow soil with an annual rainfall of 1,143 mm and annual average temperature of 15 °C. Three male and female *T. fortunei* were selected for sampling. Male and female flowers in the unexpanded flower buds were collected and labeled as Pt-ff (female flower) or Pt-mf (male flower). The middle part of unexpanded leaves at the top of the treetop was collected (light yellow) and labeled as Pt-fl (female leaves) or Pt-ml (male leaves), and then promptly placed in liquid nitrogen. There are three biological repeats in each group.

### RNA extraction and library preparation

The isolation of total RNA from above samples were isolated according to the instruction manual of the Trizol Reagent (Invitrogen, Carlsbad, CA, USA). Nandrop 2000 (Thermo Fisher Scientific, Waltham, Massachusetts, USA) was used to detect the purity of RNA. Qubit was used to accurately quantify RNA concentration. Agilent 2100 (Agilent Technologies, Santa Clara, California, USA) accurately detected RNA integrity. After passing these quality checks, magnetic beads with Oligo (dT) were enriched for eukaryotic mRNA. Subsequently, fragmentation buffer was used to break the mRNA into short fragments, which was used as a template to synthesize a strand of cDNA with a 6-base random primer. Then, double-stranded cDNA was synthesized, purified, and subjected to end repair, poly-(A) tail synthesis, and ligation to the sequencing link. Fragment size selection was conducted using AMPure XP beads. The second strand of the cDNA containing U was degraded using USER enzymes and the strand orientation of the mRNA was retained. After the prepared library was tested, sequencing was performed on Illumina HiSeq 2500 system machine. There are a total of 12 samples, and each sample is constructed separately for library sequencing.

### Evaluation, assembly, and annotation of raw data quality

The raw sequencing data was quality-controlled; low-quality reads and unknown bases > 1% were removed using the Trimmomatic tool^35^. Trinity software^36^ was used for clean reads assembly. Unigene sequences (longest transcript) were aligned with the NCBI NR^37^, Swiss-Prot^38^, Gene Ontology (GO)^39^, COG^40^, EuKaryotic Orthologous Groups (KOG)^41^, eggNOG^42^, and Kyoto Encyclopedia of Genes and Genomes (KEGG) databases^43^.

### Gene expression quantification, differential analysis, and functional enrichment

The reads obtained from sequencing were compared with the unigene library using Bowtie software^44^. Expression levels were estimated according to the comparison results and RSEM^45^. Pearson's correlation coefficient was used as an indicator for evaluating biological repetitive correlations^46^. R software (https://www.r-project.org/) was used to calculate the correlation coefficients, plot the correlation heatmap, and conducted a principal component analysis (PCA). Use DESeq^47^ software to standardize the number of Unigene counts in each sample (basemean value is used to estimate expression), calculate the difference multiple, and use NB (negative binomial distribution test) to test the difference reads significance, and finally screen differentially expressed gene (DEG) according to the difference multiple and the difference significance test results. DEG screening threshold was set to |fold change| ≥ 2 and FDR < 0.01. Perform hierarchical clustering (hcluster, hierarchical clustering) analysis on DEG, annotate the function of the database with DEG, and perform functional enrichment analysis on DEG using software topGO^48^ and KOBAS^49^.

### RT-qPCR verification

Real-time quantitative PCR (RT-qPCR) amplification was conducted using an SYBR Premix Ex TaqTM II kit. Twelve genes were selected; the actin gene was used as the reference gene, Primer Primer5 software was used to design primers (refer to Table 4 for the primer list). All samples reported in the transcriptome were quantitatively verified; each sample had three biological and three technical replicates. The first strand of the cDNA fragment was synthesized from total RNA. The RT-qPCR reaction conditions were as follows: preheating at 95 °C for 30 s, 40 cycles at 95 °C for 5 s, and annealing at 60 °C for 34 s. Relative expression levels were calculated using the 2^−ΔΔCt^ method^50^.

**Table 4:** List of primers used in this study.

No.	Gene symbol	GeneBank	Forward primer	Reverse primer	Product length(bp)	Ta (℃)
1	CHD1	TRINITY_DN61762_c0_g1	GAGTGAAGAGGAGCCATTTG	GGCATTCCCACCATAAGTATC	61	60
2	STPK	TRINITY_DN81704_c4_g4	AGTACCATCACCCATGACC	CGAAGATGAGTGCTGTTGT	83	60
3	P450 716B1	TRINITY_DN82812_c2_g2	CCAGTTCTACTTGGCTAGTC	CATTGATAGTGTGAGCAAACC	64	60
4	UPF0136	TRINITY_DN70437_c2_g4	TCTATGTATGCACTGGGCTTC	ATCAACAGCCACATTCTCG	90	60
5	Cytokinin dehydrogenase 6	TRINITY_DN64789_c0_g1	AATGGAGGATGGGTATTCACA	GGGACATGATAGAGCACCGA	89	60
6	Thump	TRINITY_DN73064_c0_g1	ATATGGGCAATTATTAGCTGGT	AGTGAGTAGGATTCTAGTGCTT	60	60
7	FT	TRINITY_DN75953_c2_g6	GCACATGAAGACATGAACCT	GTAACTGCTTGGGATGATGAC	74	60
8	PI	TRINITY_DN70832_c3_g1	TTACCGGAAACTCAGCAGTC	TATCCAAGCTCCAGCTCCCTTA	116	60
9	AG	TRINITY_DN69681_c4_g1	AGTACAGCAAGTAGGTATTGTG	ATGGTAATAGTTTCGGGAATCG	79	60
10	AG9	TRINITY_DN81743_c0_g1	ATGGAAAGTAATGGAAACCAGT	GGATTATGATGTGTGAAGCTCT	132	60
11	Sterile apetala	TRINITY_DN72937_c1_g3	CTCGCCACAGTGCTAAAC	CAGGGCATTGGTGGATTC	126	60
12	FLC	TRINITY_DN71780_c5_g1	AGGCTAAGGAACTGTCGAT	CAGTGGGCGAGAACATGA	63	60
13	Actin	TRINITY_DN66424_c1_g2	TGAATCTGGTCCATCCATTGTC	AGAACATACCATAACCAAGCTC	60	60
